# Medullary Sarcoma of the Spleen

**Published:** 1867-09

**Authors:** L. Woodruff

**Affiliations:** Alton, O.


*Medullary Sarcoma of the Spleen.* By L. Woodruff, M. D., Alton, O.

June 29, 1866, was called to see Mrs. M. H., aged 28 years, the mother of five children. She complained of a "lump" in her left side, which could be distinctly felt below the ribs, and was occasionally painful, and slightly tender. My diagnosis was an enlarged spleen, and gave her a very mild mercurial, and iron and quinia. The tonics were continued at intervals during the summer without any perceptible diminution of the tumor, or serious impairment of the general health, until the 25th of Octber, when she had an attack of fever, accompanied with great enlargement, extreme lancinating pains, and much tenderness of the tumor.

The fever soon gave way under appropriate treatment. Now the patient exhibited the peculiar sallow paleness of complexion characterizing the cancerous cachexia. This, together with the lancinating pain, and the obstinacy with which it had resisted all treatment convinced me of the malignant character of the tumor. Prof. Hamilton, of Starling Medical College, saw her in the latter part of November, and Dr. Gay, of Columbus, in January, both of whom were convinced of its cancerous nature.

A supporting and anodyne course of treatment, and nutritious diet were continued until March last, when an Eclectic took charge of the case, assuring the patient that he could "*cure*" her, and in the stereotyped language of the charlatan, assured her that "*if she had been treated right in the beginning, she would have been well long ago.*" The cancerous vulture of incurable disease still fed upon the vitals of the too confiding sufferer, in spite of the persevering use of podophyllin, spearmint, and catnip. The vital forces rapidly failed, and on the 20th of June, 1867, one year after the first appearance of the tumor, in an effort to rise from her bed, she had fatal syncope, and soon expired.

Autopsy twenty hours after death. Emaciation, in fact almost entire absence of fatty tissues. Abdomen distended with fluid, and in left side a lobulated tumor. On opening the abdomen, about two gallons of straw colored fluid were discharged. The tumor occupied the left side, crowding the intestines to the opposite side. Tumor firmly adherent to transverse colon, pelvis of left kidney, pancreas, stomach posterior wall of the abdomen, and in fact to all the surrounding parts.

Tumor, perhaps eighteen inches in circumference, firm, nodulated and cartilaginous in appearance. In breaking up the adhesion, a quantity of matter of the consistence and white appearance of cream, the result of softening. The tumor consisted of a "dull, opaque, softish matter, bearing a resemblance to brain." Dissection showed the tnmor to have originated in the spleen, to which it was attached by a short pedicle, an inch in diameter. The spleen was two or three times its natural size, in a state of degeneration; the healthy structure had been replaced by the unhealthy until about one-fourth of that organ presented the same appearance as the tumor. Right kidney healthy; left atrophied, apparently from pressure. In the right lobe of the liver, two round masses, two inches in diameter, were found, resembling the tumor in appearance, but in no way connected with it; remainder of the organ healthy. Omentum entirely destitute of fat, firmly attached to tumor, and vessels injected. Uterus and its appendages were healthy.

